# Intrinsic individual variation in daily activity onset and plastic responses on temporal but not spatial scales in female great tits

**DOI:** 10.1038/s41598-022-22935-1

**Published:** 2022-10-26

**Authors:** Marjolein Meijdam, Wendt Müller, Marcel Eens

**Affiliations:** Department of Biology, Behavioural Ecology and Ecophysiology Group, University of Antwerp, Universiteitsplein 1, Wilrijk, 2610 Antwerp, Belgium

**Keywords:** Behavioural ecology, Animal behaviour

## Abstract

In a variety of species, individuals appear to be consistent in the daily timing of their activity onset. Such consistent among-individual differences can result from both intrinsic factors, as individuals may e.g. differ genetically, and extrinsic factors, as the environment may vary on spatial and temporal scales. However, previous studies typically did not differentiate between their respective contributions on individual variation in the timing of activities. Here, we repeatedly measured the onset of activity in female great tits (*Parus major*) on consecutive days during the egg laying phase of the breeding season in four consecutive years. Subsequently, we used a variance partitioning analysis in order to determine which part of the total variation could be attributed to intrinsic (female identity) and extrinsic (nest box identity) factors. Overall, 27% of the total variation could be attributed to female identity. In addition, we found temporal variation in the activity onset, indicating that individuals can plastically adjust their timing. Yet despite their general ability to change the timing of activities over time, spatial environmental factors did not contribute significantly to the observed variation. Individuals may choose a habitat that matches the preferred timing of activities, or might not benefit from adjusting their timing to environmental factors that might vary on spatial scales.

## Introduction

The timing of the daily active period differs tremendously among species, which has among others led to a categorisation of species being diurnal, nocturnal or crepuscular. From a functional or ecological perspective, the key drivers of this temporal niche partitioning in the timing of the active period are thought to be reduced competition among species and/or avoidance of predation^1–3^. Also within species consistent differences among individuals in the timing of the onset of the active period have been reported. Although among individual variation is less pronounced than the variation among species (see^4^ for an overview in birds), multiple studies have shown the existence of consistent individual differences in the timing of their active period, so called ‘chronotypes’^5^, in a variety of species. For example, free-ranging pearly razorfish (*Xyrithchys novacula*), Arctic ground squirrels (*Urocitellus parryii*) and blue tits (*Cyanistes caeruleus*) are repeatable in their activity onset and offset^4,6,7^. Yet despite increasing evidence, not only the functional consequences, but also the underlying causes of this individual variation are largely unknown^8^. Identifying the factors that underlie the above-mentioned consistent differences is therefore crucial for a deeper understanding of the ultimate causes of intraspecific variation in the timing of activity.

There are several possible underlying drivers that could lead to consistent differences in the timing of activities among individuals. On the one hand, intrinsic characteristics in the functioning of the biological clock may differ among individuals^9^. For some species, it has been shown that genetic differences among individuals are related to differences in the functioning of the internal biological clock and that certain characteristics of the biological clock have a hereditary component^8,10–14^. The biological clock, in turn, can influence the timing of activity and hence contribute to individual variation, although the exact mechanisms still need to be clarified^5^. As a matter of principle, maternal effects, epigenetic effects or developmental plasticity may be responsible for intrinsic differences in the functioning of the biological clock as well, although little is known on this subject yet.

On the other hand, consistent differences in the environment in which individuals find themselves could equally lead to consistent differences in the timing of behaviour. These extrinsic factors may act on temporal as well as on spatial scales. On a temporal scale fluctuations in food availability have been shown to influence the timing of the dawn song in male birds^15–17^ and higher temperatures are related to earlier activity onset in blackbirds (*Turdus merula*)^18^, but cause a slightly later onset of activity in great tits (*Parus major*)^19^. Because environmental factors can change over time it is likely that individuals are more consistent in the timing of activity in the short term than in the long term^4,20^, hence consistent individual differences may contain a temporal component.

On a spatial scale anthropogenic stressors, predation risk and (again) food availability could be key environmental drivers causing individual variation in activity patterns. Anthropogenic stressors, like artificial light at night and noise pollution can disrupt sleep and normal circadian rhythms and as a consequence affect activity patterns. Artificial light at night is related to earlier activity onset in great tits^21^ and blackbirds^18^ and noise pollution leads to an earlier activity onset in great tits^22^. Fear of humans and perceived predation risk may affect activity patterns as well. In areas with high human disturbance mammals shift their active phase and become relatively more nocturnal^23^, while higher predation risk is related to longer sleep duration in great tits^24^. As environmental factors vary on a spatial scale, it can be expected that individuals that are repeatedly measured in one location are more consistent in their behaviour than individuals that have moved^20,25^. Furthermore, food availability may not only change on a temporal scale, but can also differ spatially such as among territories, implying that spatiotemporal variation in environmental factors (partly) shapes activity patterns.

Spatiotemporal variation in environmental factors may thus cause consistent differences among individuals in the timing of activity onset that are not attributable to intrinsic characteristics. Although there are several indications that both intrinsic differences among individuals in the functioning of the biological clock and spatiotemporal variation in environmental factors can in interplay cause consistent differences in behaviour among individuals^4,20,25^, their relative importance in determining activity patterns is yet unknown.

Phenotypic variation is likely caused by processes operating at multiple levels, i.e. a certain phenotype is expressed by an individual from a given population at a given moment of time, and this necessitates to consider hierarchical structures^26^. Partitioning of variances can provide information on the different variance components and also allows to estimate their repeatability, which may improve our understanding of the evolutionary potential of a certain behavioural trait, here chronotypes. Yet, behavioural ecologists often try to relate an individual’s mean trait value to fitness parameters like reproductive success, ignoring possible variation in labile traits within individuals, and how trait expression differs among hierarchical levels. Both behavioural traits and fitness parameters may co-vary with (changes in) the environment, which may result in the reporting of non-causal relationships and relationships that hold true on the within-individual level, but not on the among-individual level^27^. Revealing the relative contributions of intrinsic and extrinsic sources of variation, especially by distinguishing between spatial and temporal variation in the environment, may thus help to understand which factors shape behaviour and how to correctly measure it.

Here, we use longitudinal data to investigate the sources of among individual variation in activity onset in a nest box breeding population of great tits. To this end, we measured the timing of the first emergence from the nest box of female great tits during the egg laying phase of the breeding season in four consecutive years. As female great tits often breed in multiple years it is possible to determine repeatability of activity onset both in the short term (i.e. within years) and in the long term (i.e. between years). This provides insight into the importance of temporal variation in environmental effects in causing consistency in the timing of activity onset. Furthermore, environmental effects may add a spatial component, as some females breed multiple years at the same location (i.e. in the same nest box), while others switch from breeding location (i.e. they breed in a different nest box). Additionally, there are nest boxes which were used by multiple females over the years. This individual-based spatiotemporal information allows for the use of a variance partitioning analysis, enabling us to determine which part of the variation in activity onset in the population can be attributed to differences among individuals (i.e. intrinsic differences) and differences among nest boxes (i.e. extrinsic/environmental differences) in which individuals are measured^20,25,28,29^.

## Materials and methods

All data were collected in a nest box breeding population of great tits, located in Wilrijk (Antwerp), Belgium (51°09′46.1"N, 4°24′13.3"E), during the breeding seasons of 2018, 2019, 2020 and 2021. In the study area about 170 nest boxes are available in trees at a height of about two meters. The population (covering ± 2.4 km^2^) is located in a suburban area with a large amount of environmental variation in terms of light and noise pollution^30^, vegetation type and cover and ground surface hardening. Yearling female great tits are reproductively mature and have a life expectancy of 12 to 21 months, with a maximum of seven to 10 years^31,32^, so they often breed in multiple years. During the egg laying phase of the breeding season females lay one egg each morning for 5 to 13 days in a row in our population. In the morning females leave the nest box for the first time after laying the egg^33^. In order to enable individual recognition all individuals were caught in the nest box during roosting in winter or during chick feeding in the breeding season and equipped with a PIT tag (passive integrated transponder; EM4102, 125 kHz, Eccel Technology Ltd, Aylesbury, UK) and a unique combination of colour rings. During the breeding season all nest boxes were checked regularly. When the nest building was completed nest boxes were checked every day, so the lay date of the first egg was known for all pairs. Emergence times (i.e. the first time a female leaves the nest box in the morning) were measured on multiple consecutive days during the egg laying phase. To minimise disturbance, nest boxes were not checked after the first egg was laid until all data were recorded. In our population females can have up to two broods per year, but in this study all emergence times were measured during the egg laying phase of the first breeding attempt only.

In order to obtain data on as many females as possible, emergence time from the nest box was measured using three different devices: SongMeters (SongMeterTM SM2 + ; Wildlife Acoustics, Inc, U.S.), radio-frequency identification (RFID) loggers (EM4102 data logger, Eccel Technology Ltd, Aylesbury, U.K.) and infrared sensitive cameras (Pakatak PAK-MIR5, Essex, UK, ^34^). SongMeters were placed on top of the nest box, with one microphone inside and the other microphone outside the nest box. Sound was recorded from 04:00 to 08:00 a.m. CET during the winter time period. After the clock changed to summer time sound was recorded from 03:00 to 08:00 a.m. CET. When females leave the nest box in the morning the sound of their wings can often be heard as well as their claws on the nest box^35^ and a change in air pressure when the female passes through the opening of the nest box. Avisoft SASLab Pro 5.2.14 was used to determine emergence time^36^. RFID readers register PIT tagged individuals when they fly through the two antennas, which were placed around the nest box opening. Both the unique PIT tag number and the time of leaving/entering the nest box was saved (for more details see^37^). Infrared sensitive cameras were installed under the lid of the nest box, pointing downwards. The cameras recorded immediately after installation at least 2 h before sunset and were switched off on collection the next morning at least 2 h after sunrise^38^.

From the total of 1076 observations 5 datapoints were collected using the infrared cameras (1 in 2018, 4 in 2021), 49 using the RFID loggers (9 in 2018, 40 in 2020) and all remaining data via SongMeters (see also^39^ for more details on the data selection process). We removed one datapoint from the dataset as it was an outlier. The emergence time was more than an hour earlier than all other datapoints. In total, data were collected on 162 females in 118 different nest boxes in 4 consecutive years. Within years emergence times were repeatedly measured on one to eight consecutive mornings per female (3.60 ± 1.22 times, mean ± s.d.), within one nest box. Between years females can switch nest boxes for breeding. Females were measured in one to four different years, in up to three different nest boxes (see Table 1 for a detailed overview of sample sizes for females). For nest boxes, emergence times were measured in one to four different years, with up to three different females (see Table 2 for a detailed overview of sample sizes in nest boxes). All emergence times were determined relative to sunrise on the day of measurement (negative = before sunrise, positive = after sunrise). Temperature data (measured every 30 min) were retrieved from a local weather station at the Antwerp international airport nearby our study population (± 5 km) via: https://www.wunderground.com/history/daily/be/antwerp. In our statistical analyses we used the temperature that was measured closest to sunrise.

**Table 1:** Overview of the number of different nest boxes and years in which females were measured.

Number of different years in which a female was measured	Number of different nest boxes in which a female was measured			
	1	2	3	4
1	60			
2	40	32		
3	9	12	2	
4	2	2	3	0

For example, there were 12 individuals that were measured in 3 different years in 2 different nest boxes (i.e. they moved once) and 9 individuals that were measured in 3 different years in only 1 nest box (i.e. they did not move).

Repeated measures within years are not included in this table.

**Table 2:** Overview of the number of years in which a nest box was occupied and the number of different females that were measured inside a nest box.

Number of different years in which emergence times were measured in a particular nest box	Number of different females that were measured in the nest box			
	1	2	3	4
1	8			
2	10	39		
3	6	39	4	
4	2	8	2	0

For example, there were 2 nest boxes that were occupied in 4 different years with 3 different individuals, and 8 nest boxes that were occupied in 4 different years with only 2 different individuals.

Repeated measures within years are not included in this table.

### Statistical analyses

All statistical analyses were performed in R 4.1.1^40^. We used a linear mixed model with emergence time as the response variable and a polynomial date (mean-centred within years) effect up to the second order, the temperature at sunrise (mean-centred within years), year (as categorical variable) and age of the female (yearling breeder versus older birds [> 2 years]) as fixed effects. Female identity (FemaleID), nest box identity (NestID), the unique combination of FemaleID and the year (FemaleID_Year), and the unique combination of FemaleID and NestID (FemaleID_NestID) were included as random effects. FemaleID_Year was included in the model to be able to determine the variation in emergence times among years within individuals. FemaleID_NestID explains variation among individuals in how their emergence times change in response to different nest boxes and can thus be interpreted as differences among females in their plasticity with regard to emergence times (although part of this variation may also be explained by environmental changes in the surroundings of the nest boxes between years; see “Discussion”). We used the spaMM package^41^ to obtain 95% confidence intervals for all fixed and random effects by 1000 paramatric bootstrapping simulations.

We calculated the relative contribution of each random effect to the total variance (i.e. variance partitioning), including the 95% confidence intervals, based on this model. Following^42^, we calculated the short term (= within years) (Eq. 1) and long term (= between years) repeatability (Eq. 2) for emergence times.

1 $${R}_{shortterm}=\frac{{V}_{FemaleID} + {V}_{FemaleID\_Year}}{{V}_{FemaleID} + {V}_{NestID} + {V}_{FemaleID\_NestID} + {V}_{FemaleID\_Year} + {V}_{Residual}}$$

2 $${R}_{longterm}=\frac{{V}_{FemaleID} }{{V}_{FemaleID} + {V}_{NestID} + {V}_{FemaleID\_NestID} + {V}_{FemaleID\_Year} + {V}_{Residual}}$$

As FemaleID_NestID explains differences among individuals in plasticity, it contributes to the variation that is explained by intrinsic differences among individuals. Therefore, we also calculated the combined effects of FemaleID and FemaleID_NestID relative to the total variance (Eq. 3).

3 $${R}_{intrinsic}=\frac{{V}_{FemaleID} + {V}_{FemaleID\_NestID}}{{V}_{FemaleID} + {V}_{NestID} + {V}_{FemaleID\_NestID} + {V}_{FemaleID\_Year} + {V}_{Residual}}$$

In order to compare the repeatability of emergence times between individuals that moved between nest boxes to breed in and individuals that did not move, we created two subsets. Linear mixed models were created for two subsets, which included the same fixed effects as described above, but this time only FemaleID and FemaleID_Year were included as random effects. Again we used the spaMM package to calculate the long term repeatability of moved (n_females_ = 49) and not-moved individuals (n_females_ = 69), and used 1000 parametric bootstrapping simulations to quantify 95% confidence intervals^41^.

### Ethical note

This study was approved by the ethical committee of the University of Antwerp (ID numbers: 2016–87 and 2018–50) and was performed in accordance with Belgian and Flemish laws regarding animal welfare, adhered to the ASAB/ABS guidelines for the use of animals in behavioural research and teaching, and complies with ARRIVE guidelines. The Royal Belgian Institute of Natural Sciences (KBIN) provided ringing licenses for all authors and technicians. Handling time was minimized as much as possible. All other methods described above are non-invasive.

## Results

**Figure 1:**
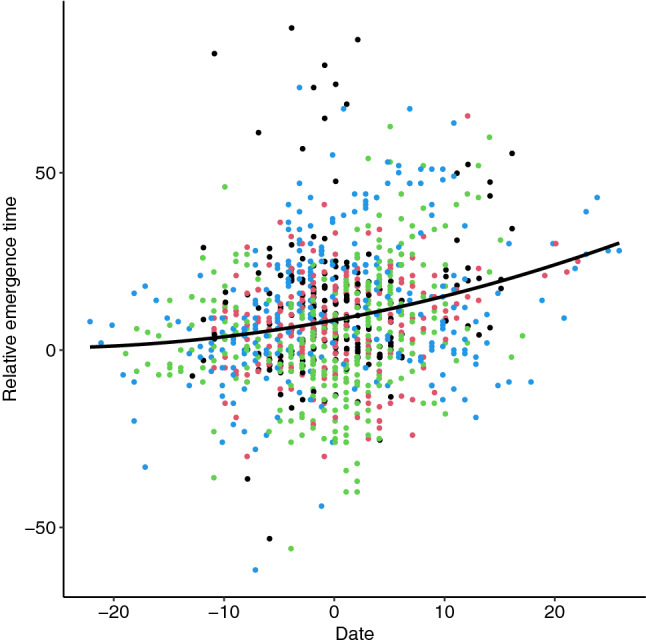
Date (mean-centred within years) positively affects relative emergence time (in minutes; negative = before sunrise, positive = after sunrise). Black = 2018, red = 2019, green = 2020, blue = 2021.

On average female great tits emerged from the nest box 8.9 min after sunrise (s.d. = 18.0 min, min = − 62 min, max = 91 min). Compared to 2018, females emerged 5.9 min earlier from the nest box in 2020 and 2.8 min later in 2021 (Table 3). Emergence times were affected by date (Fig. 1, Table 3). Later during the breeding season emergence times became later relative to sunrise. Furthermore, older females (> 2 years) were slightly, but significantly, earlier than yearling females.

**Table 3:** Results from linear mixed effects model with emergence time (in minutes relative to sunrise) as response variable.

Fixed effect	β	T	Lower 95% CI	Upper 95% CI
Poly(centred date)1	147.86	5.66	135.00	193.10
Poly(centred date)2	60.65	2.52	47.64	110.77
Centred T sunrise	− 0.30	− 2.06	− 0.45	0.11
Year 2019	− 2.06	− 1.08	− 3.96	0.38
Year 2020	− 5.92	− 2.82	− 8.00	− 3.51
Year 2021	2.75	1.10	0.08	5.11
Older/yearling	− 4.24	− 2.66	− 5.64	− 2.30

Random effect	σ^2^		Lower 95% CI	Upper 95% CI
FemaleID	79.46		58.42	104.64
NestID	19.42		1.74	36.45
FemaleID_Year	53.19		53.67	91.30
FemaleID_NestID	19.62		− 0.95	53.11
Residual	119.40		107.80	131.50

Date and temperature (T) at sunrise were mean-centred within years. 95% confidence intervals are calculated with parametric bootstrapping.

**Figure 2:**
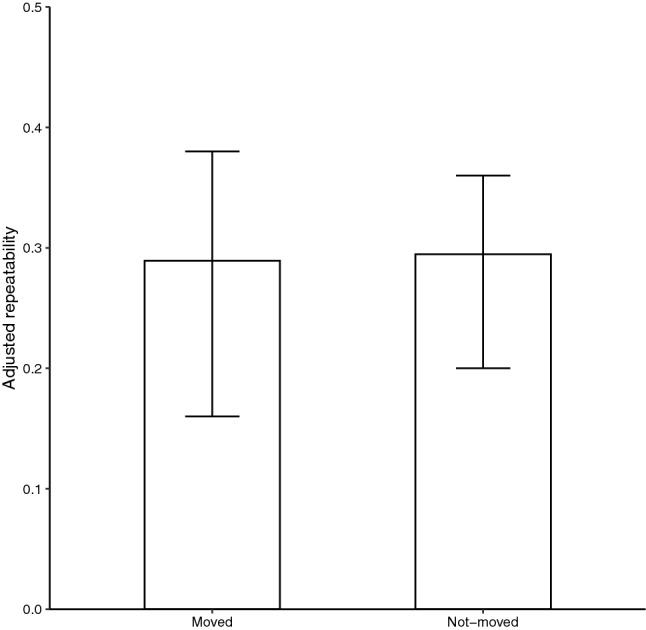
The long term repeatability of emergence time for females that did not move between nest boxes and females that moved (adjusted for year, age, date, date^2^ and temperature at sunrise). 95% confidence intervals are indicated.

Both the short and long term repeatability for emergence time were significant, although the long term repeatability was lower than the short term repeatability (R_short term_ [95% CI] = 0.46 [0.39, 0.59]; R_long term_ = 0.27 [0.16, 0.34]). NestID and FemaleID_NestID did not explain significant parts of the total variation (Table 4). FemaleID and FemaleID_NestID together explained 34% of the total variation (R_intrinsic_ = 0.34 [0.24, 0.43]). The long term repeatabilities of emergence time for females breeding in the same nest box and females that moved were very similar (R_moved_ [95% CI] = 0.29 [0.16, 0.38]; R_not-moved_ = 0.29 [0.20, 0.36]; Fig. 2; Tables S1,S2).

**Table 4:** The relative contribution of random effects to the total variance.

Random effect	Variance partitioning
FemaleID	0.27 [0.16, 0.34]
NestID	0.07 [− 0.008, 0.12]
FemaleID_Year	0.18 [0.17, 0.31]
FemaleID_NestID	0.07 [− 0.01, 0.19]
Residual	0.41 [0.32, 0.41]

95% confidence intervals are calculated with parametric bootstrapping and are shown between brackets.

## Discussion

In this longitudinal study we investigated among individual variation in activity patterns and how consistent differences among individuals in the timing of behaviour arise. To this end, we used a variance partitioning analysis. Emergence time as measured during the egg laying phase of the breeding season had a significant intrinsic component, being repeatable both within and between years. Among individual differences in plastic response to nest box changes (= FemaleID_NestID) did not contribute significantly to the total variation. The repeatability that was caused by intrinsic differences between individuals was thus mainly explained by among individual differences (= FemaleID). We also identified a temporal component in activity onset (= difference between short and long term repeatability), while environmental effects at the spatial scale (= NestID) appeared to be of less importance.

### Temporal effects

We found a considerable difference between short and long term repeatability. This indicates that on short time scales the environment is more stable than on longer time scales, which causes individuals to behave more consistent in the short term than in the long term. This may be due to factors like weather conditions or prey abundance which often change slowly and are therefore more alike on short time scales. Furthermore, temporal autocorrelation of measurements on short time scales may also be caused by the internal state of an individual. For example, body condition typically varies little from day to day^43^. Therefore, the variance within individuals will be relatively small in the short term and as a consequence the relative contribution of among-individual variance to the total variation (= repeatability) will be larger^20,44^.

As most studies do not take temporal variation in the environment into account when studying repeatability of activity onset, many studies on repeatability of activity onset have reported estimates that are similar to or exceed our short term repeatability estimate^7,18,22,45–48^. However, Schlicht et al. (2014) tried to differentiate between short and long term repeatability of activity onset in blue tits (here mean emergence time within years for each female was used to calculate long term repeatability). Between years a non-significant repeatability estimate of 0.15 was found ^47^. Furthermore, Stuber et al.^49^ reported a repeatability estimate of 0.13 for activity onset in great tits ^49^. Here, no statistical distinction was made between short and long term repeatability, but the interval between measurements was much larger than in most other studies (months or a year instead of days). In Arctic ground squirrels repeatability estimates of activity onset decreased when the number of consecutive sampling days increased^4^. Altogether, this suggests that temporal autocorrelation of datapoints can influence repeatability estimates.

In captivity, short term repeatability of activity onset for male great tits was 0.40^19^, which is fairly similar to our short term estimate. However, in captivity temporal variation in the environment is much smaller, so the repeatability is not expected to decrease as much over time. The lower long term repeatability estimate we found in free-living great tits seems to further underline that individuals may plastically adjust the timing of activity onset to environmental factors that vary on a temporal scale (although the sexes may differ in repeatability as well).

### Spatial effects

Spatial variation in the environment did not explain a significant proportion of the total variation in emergence time. In another great tit population and in blue tits there were also no consistent differences among nest boxes in activity onset^48,49^. This result was nevertheless unexpected, as for example artificial light at night and noise pollution levels, which are known to affect activity onset^18,21,22^, vary throughout our suburban study population^30^. One possible explanation for the absence of a spatial component may be that when great tits move between nest boxes, they very often stay in the vicinity of their previous nest box, so that the environmental differences between the previous nest box and the new one may be small (only one individual moved more than two nest boxes further). It is therefore possible that environmental effects act on a larger spatial scale, and remain hence undetected in our study. However, we think that this might rather be unlikely, given the substantial differences already being present at a local scale. A visual inspection of emergence times on a map of our nest boxes indeed did not reveal obvious spatial patterns in emergence times, e.g. in the context of urbanisation. Nest boxes may also (partly) shield individuals from environmental factors like artificial light and noise pollution^50^, particularly in females that sleep in their nest box during the entire breeding season. Furthermore, phenotype habitat matching^51^, where individuals settle in certain habitats in a non-random way, could explain the minor contribution of spatial variation to the total variation. For example, individuals that are more easily disturbed by anthropogenic stressors, such as artificial light at night and noise pollution, may only choose nest boxes with low levels of these stressors. Such non-random settlement would cause a limited amount of environmental variation between the different nest boxes in which an individual chooses to breed. Another possibility may be that certain factors force individuals to become active within a specific time window, for example to avoid competition for food or to minimise predation risk, which makes it impossible to adjust their behaviour to the local conditions. Finally, it is possible that spatial variation in the environment among nest boxes is not constant over time, but that it fluctuates among years, so that environmental effects on a spatial scale (NestID effect) do not contribute substantially to the variation. Instead, this spatiotemporal variation may show up in the differences among individuals in their plastic response to different nest boxes (FemaleID_NestID effect; see below).

### Individual differences in plastic response

Differences among individuals in their temporal plasticity (= change in activity onset in function of nest box changes) did not explain a significant part of the variation observed. This is intriguing, because previous research on great tits showed a large variation in how individuals changed their emergence time after exposure to artificial light at night inside a nest box^21^. As nest boxes differ in light exposure, one could have expected to find differences among individuals in their plasticity. However, as mentioned above, emergence times were only measured for females that slept inside a nest box and nest boxes can shield individuals from light exposure^50^.

In order to determine which part of the total variation in activity onset was caused by intrinsic differences among individuals we determined the combined effects of FemaleID and FemaleID_NestID relative to the total variance. However, in our dataset, nest box changes occurred across years, so that there is a significant temporal component as well. That is, part of the variation explained by differences among individuals in their plastic response to different nest boxes may actually be due to spatial fluctuations between years in the environment. Our great tit population is suburban and therefore very dynamic (e.g. due to changing construction sites) and spatial patterns of anthropogenic stressors may have changed between years. Furthermore, females can change not only their nest box, but also their partner between years (83% of the movements between nest boxes, where the males were known in both nest boxes, were associated with a change of partner). Before females leave the nest box in the morning they often communicate vocally with their partner^52,53^ (audible on SongMeters). Therefore, the timing or the quality of the partner’s dawn song may affect female emergence times. However, Steinmeyer et al.^54^ found a correlation between awakening times of blue tit partners that were standardised within days per sex, so that relatively early males mated with relatively early females, but no significant correlation was found between the absolute awakening times (i.e. not standardised), which may suggest there is no direct effect of the male’s awakening time on the female’s emergence time^54^. Including male identity in our model might have revealed whether there may be other mechanisms by which the male affects female emergence time (e.g. male quality or song characteristics), but unfortunately, we did not have enough data to estimate the male’s contribution properly.

Finally, despite our large dataset on female emergence times, it has to be taken into account that the number of females that moved was relatively low. Therefore, it is possible that our dataset did not contain enough information to properly estimate differences among individuals in their temporal plasticity.

### Within individual variation

In this study emergence times were determined only during the egg laying phase of the breeding season and during this period females may be more consistent in the timing of their activity onset than during other periods. As the daily timing of egg laying seems to be under control of the biological clock^55,56^ and females always leave the nest box after laying the egg^33^, there may be constraints to lay and thus to emerge earlier. This may result in smaller variation within individuals during the egg laying phase than during other periods and consistency of activity onset may thus be larger during the egg laying phase.

### Chronotypes – consistent daily activity patterns in a changing world

We performed this study in a great tit population that is located in a suburban area with a large amount of environmental variation, e.g. in terms of light and noise pollution. We therefore expected to find an effect of the nest box reflecting this spatial variation (see also^18,57^). However, we did not find any indications that spatial variation affected emergence times in female great tits sleeping in nest boxes. This suggests that great tits might be resilient to light and noise pollution—at least as long as they sleep in nest boxes or similarly sheltered nests, since experimental studies showed that exposure to both light and noise pollution affect the start of activity in great tits^22,38,58^. Whether urbanisation poses a challenge for timing in great tits may thus depend on their choices of nest locations and the availability of cavities to roost in.

Not only urbanisation, but also climate change may affect the timing of activity in great tits. In recent years, egg laying dates in great tits have significantly advanced (e.g. in Belgium^59^) and earlier laying dates are associated with late sunrise and late onsets of activity. Individuals may thus have to adjust the timing of activity onset so that they are not restricted by the shorter daylengths, e.g. in the time they need to forage. Here, we found relatively large amounts of residual variation and variation within individuals between years, which indicates that there might be sufficient plasticity in emergence times. However, at some stage there may be limitations to becoming active earlier, for example because of problems with vision in the dark, so for how long great tits can keep track of advancing spring conditions for egg laying remains to be seen.

## Conclusion

Repeatability of activity onset has been determined many times in several bird species. However, to the best of our knowledge, never before have temporal and spatial variation in the environment been taken into account at the same time in these analyses. Neither have among-individual differences in the plasticity of timing been taken into account. Here, we show that emergence time in free-living female great tits has a significant intrinsic component and may hence be subjected to both sexual and natural selection pressures^5^. Surprisingly, female great tits did not adjust their behaviour to environmental variation on spatial scales. At the same time we show that the timing of activity onset varies over temporal scales and that the variation within individuals is relatively large. Activity onset in female great tits is thus a rather plastic trait, which may be sensitive to the prevailing environmental conditions, although the exact components still need to be identified.

## Supplementary Information

Supplementary Tables.

## Data Availability

The dataset analysed during the current study is available from the corresponding author (Marjolein Meijdam) on reasonable request.
