# Supplementary material for: Intrinsic individual variation in daily activity onset and plastic responses on temporal but not spatial scales in female great tits
Source: Sci Rep. 2022 Oct 26;12:18022. doi: 10.1038/s41598-022-22935-1 (PMC9605954; doi:10.1038/s41598-022-22935-1)
Supplement: Supplementary file 1 — Supplementary Tables. [file 41598_2022_22935_MOESM1_ESM.pdf]

Supplementary Material

Intrinsic individual variation in daily activity onset and plastic responses on temporal but not spatial scales in female great tits

Marjolein Meijdam[1]*, Wendt Müller[1], Marcel Eens[1]

[1]Department of Biology, Behavioural Ecology and Ecophysiology Group, University of Antwerp, Wilrijk, Belgium

Table S1: Results from linear mixed effects model which included only females that moved between nest boxes, with emergence time (in minutes relative to sunrise) as response variable. Date and temperature (T) at sunrise were mean-centred within years. 95% confidence intervals are calculated with parametric bootstrapping and are shown between brackets.

Fixed effect	β	T	Lower 95% CI	Upper 95% CI
Poly(Centred Date)1	112.60	4.58	85.1	140.3
Poly(Centred Date)2	61.95	2.87	45.57	105.70
Centred T sunrise	-0.27	-1.24	-0.63	0.20
Year 2019	0.66	0.17	-2.93	4.27
Year 2020	-3.37	-0.83	-6.91	-0.32
Year 2021	9.82	2.02	5.50	13.06
Older/Yearling	-6.28	-2.04	-9.07	-4.02
Random effect	σ^2		Lower 95% CI	Upper 95% CI
FemaleID	75.49		50.81	104.70
FemaleID_Year	101.50		93.4	151.1
Residual	83.91		69.93	98.48

Table S2: Results from linear mixed effects model which included only females that did not move between nest boxes, with emergence time (in minutes relative to sunrise) as response variable. Date and temperature (T) at sunrise were mean-centred within years. 95% confidence intervals are calculated with parametric bootstrapping and are shown between brackets. Note: the estimate for FemaleID_Year falls outside of the confidence interval. However, using the estimate for the calculation of the repeatability gives very similar results to using the upper limit of the confidence interval (with estimate R= 0.29 [0.20, 0.36]; with upper limit of the confidence interval R = 0.25 [0.19, 0.34]). Thus, this does not pose problems for the interpretation of the results.

Fixed effect	β	T	Lower 95% CI	Upper 95% CI
Poly(Centred Date)1	97.57	3.99	88.45	142.77
Poly(Centred Date)2	44.40	1.97	15.29	69.45
Centred T sunrise	-0.25	-1.29	-0.40	0.33
Year 2019	0.47	0.19	-2.78	3.52
Year 2020	-2.46	-0.88	-5.94	0.89
Year 2021	3.95	1.16	-0.20	8.12
Older/Yearling	-3.82	-1.79	-5.79	-0.54

Random effect	σ^2	Lower 95% CI	Upper 95% CI
FemaleID	68.11	51.18	90.32
FemaleID_Year	43.74	45.83	80.04
Residual	119.30	103.4	135.0
